# Differences in the effects of anxiolytics bromodihydrochlorophenylbenzodiazepine and fabomotizole in patients with anxiety disorders in dependence on their individually-typological features

**DOI:** 10.1192/j.eurpsy.2022.987

**Published:** 2022-09-01

**Authors:** O. Dorofeeva, M. Metlina

**Affiliations:** FSBI “Zakusov Institute Of Pharmacology”, Department Of Pharmacological Genetics, Moscow, Russian Federation

**Keywords:** anxiolytic, individually-typological features, therapeutic effects

## Abstract

**Introduction:**

Personalized approach in drug therapy is an essential line of modern psychiatry. Experimental and clinical studies of anxiolytics have shown differences in drug effects in dependence on genetically determined reactions to stress and personal features.

**Objectives:**

To evaluate of the therapeutic effects and effectiveness of bromodihydrochlorophenylbenzodiazepine and fabomotizole in dependence on individually-typological features of patients with anxiety disorders.

**Methods:**

45 patients (mean age 33,3±9,7 years) with generalized anxiety disorder (n=22) and panic disorders with agoraphobia (n=23) participated in this open-label study. 13 patients treated with typical anxiolytics bromodihydrochlorophenylbenzodiazepine at dose 2 mg daily and 32 patients treated with atypical fabomotizole at dose 30 mg daily. The duration of treatment was 14 days. Minnesota Multiphasic Personality Inventory, Psychiatric Symptoms Severity Evaluation Questionnaire and CGI-E were administered.

**Results:**

Asthenic features (high pessimism, anxiety, individualism) were revealed in 26 patients and stenic features (high impulsivity, rigidity and optimism) were revealed in 19 patients. Patients with asthenic features had tranquilo-activating effect of bromodihydrochlorophenylbenzodiazepine, whereas patients with stenic features had tranquilo-sedative effect. The tranquilo-activating effect of fabomotizole was revealed in patients with stenic features. High efficacy of bromodihydrochlorophenylbenzodiazepine was observed in patients with asthenic personality traits (χ ^2^ = 7,8), whereas in fabomotizole-in patients with stenic individual typological features (χ ^2^ = 9,1).

**Conclusions:**

Patients with stenic and asthenic features had differences in therapeutic effects and the effectiveness of anxiolytics. Personality features determine the sensitivity of patients with anxiety disorders to psychotropic drugs.

**Disclosure:**

No significant relationships.

